# Safe Pregnancy intervention for intimate partner violence: a randomised controlled trial in Norway among culturally diverse pregnant women

**DOI:** 10.1186/s12884-022-04400-z

**Published:** 2022-02-21

**Authors:** Eva Marie Engebakken Flaathen, Lena Henriksen, Milada Cvancarova Småstuen, Berit Schei, Angela Taft, Josef Noll, Lisa Garnweidner-Holme, Mirjam Lukasse

**Affiliations:** 1 Department of Nursing and Health Promotion, Oslo Metropolitan University, St. Olavs plass, PO Box 4, N-0130 Oslo, Norway; 2 Division of General Gynaecology and Obstetrics, Oslo University Hospital, Nydalen, PO Box 4950, N-0424 Oslo, Norway; 3 Department of Public Health, Faculty of Medicine and Health Science, The Norwegian University of Science and Technology (NTNU), Håkon Jarls gate 11, N-7489 Trondheim, Norway; 4 Department of Gynaecology, St. Olavs Hospital, Trondheim University Hospital, Sluppen, Postbox 3250, N-7006 Trondheim, Norway; 5 Judith Lumley Centre, La Trobe University, Bundoora,, Melbourne, VIC 3086 Australia; 6 Department of Technology Systems, University of Oslo, Kjeller, PO Box 20, 2007 Lillestrøm, Norway; 7 Department of Nursing and Health Sciences, Faculty of Health and Social Sciences, University of South-Eastern Norway, PO Box 235, 3603 Kongsberg, Norway

**Keywords:** Intimate partner violence, Antenatal care, Tablet technology, mHealth technology, Video intervention, Cultureal sensitivity

## Abstract

**Background:**

Intimate partner violence (IPV) during pregnancy is a global health problem with adverse consequences for mothers, infants and families. We hypothesise that information about IPV and safety behaviours during pregnancy has the potential to increase quality of life and the use of safety behaviours and prevent IPV.

**Methods:**

A multicentre randomised controlled trial among culturally diverse pregnant women in Norway, to test the effect of a tablet-based video intervention about IPV and safety behaviours. Women attending routine antenatal check-ups alone (baseline) were screened for violence (Abuse Assessment Screen) by responding to questions on a tablet, and randomised (1:1) by computer to receive an intervention or a control video. The intervention video presented information about IPV and safety behaviours. The controls viewed a video promoting healthy pregnancy in general. Outcome measures were assessed three months post-partum: The World Health Organization Quality of Life-BREF, the Composite Abuse Scale on violence during the last 12 months and use of safety behaviours based on a 15-item checklist. A general linear model for repeated measures was used to examine the intervention’s effect. The analyses were conducted by intention to treat.

**Results:**

Among 1818 eligible women**,** 317 reported IPV and were randomised to an intervention (157) or a control group (160). A total of 251 (79.2%) women completed the follow-up questionnaire: 120 (76.4%) in the intervention group and 131 (81.9%) in the control group. At follow-up, 115 (45.8%) women reported a history of IPV. Few women (*n* = 39) reported IPV during the last 12 months. No differences in quality-of-life domains and overall quality of life and health were found between the intervention and the control groups. We detected no differences between the use of safety behaviours or IPV frequency and severity during the last 12 months.

**Conclusion:**

Our intervention did not improve women’s quality of life, use of safety behaviours or exposure to violence. Nevertheless, a tablet-based tool may motivate women experiencing IPV to seek help and support. More research is needed regarding tablet-based interventions for women experiencing IPV, particularly culturally sensitive interventions.

**Trial registration:**

NCT03397277 registered in clinicaltrials.gov on 11/01/2018.

**Supplementary Information:**

The online version contains supplementary material available at 10.1186/s12884-022-04400-z.

## Background

Intimate partner violence (IPV) during pregnancy is a serious public health problem that affects not only the mother but also the fetus [1]. IPV can include physical, sexual and emotional violence, stalking and psychological harm by a current or former partner [2]. IPV prevalence during pregnancy varies significantly in different studies, depending on the measure used [3, 4]. Most studies report prevalence between 4 and 9% [3]. Norwegian studies show that between 1 and 5% of women report violence during pregnancy [5–7]. IPV affects women from all backgrounds, regardless of ethnicity and socio-economic status [8]. However, previous studies included few women from minority populations; hence, a knowledge gap about IPV within different migrant groups exists [5, 6].

IPV is associated with increased risk of poor health, depressive symptoms, substance abuse, injury and death [2]. Pregnancy is a particularly vulnerable period for IPV because of the great physical, emotional, social and economic changes that happen to women and families [9, 10]. IPV during pregnancy is associated with adverse outcomes for both women and their babies [2], including haemorrhage, severe acute maternal morbidity, stillbirth, small-for-gestational-age newborns, low birth weight, prematurity and early cessation of breastfeeding [1, 11–14].

The antenatal period is regarded as a “window of opportunity “ to address IPV because women are in regular contact with health professionals and may be motivated towards lifestyle changes [15]. Routine enquiry about exposure to violence is recommended in antenatal care if privacy, confidentiality, guidelines and referral services exist [16]. In Norway, midwives and general practitioners who perform routine antenatal check-ups are strongly recommended to ask about violence [17]. Despite the fact that IPV is more common than adverse maternal conditions such as preeclampsia and diabetes [3], it is still a neglected issue in antenatal care, and there is limited evidence on effective interventions to prevent IPV, and thus reduce harm from IPV during pregnancy [9, 18].

Recommended interventions for IPV in primary care settings involve addressing violence with empathic listening, providing information about safety-promoting behaviours and referring to community resources [19–21]. The main aim of interventions is not only reduction of violence but also improvement of women’s physical and psychosocial well-being, since violence reduction may be difficult to see in the short term [22, 23]. Interventions in antenatal care that involve home visiting programmes have been shown to be beneficial against IPV [24, 25]. Shorter educational and counselling interventions have also been conducted, indicating that less comprehensive interventions may result in the use of safety behaviours and reduced IPV levels [19, 26, 27]. Studies have examined the use of mobile health (mHealth) devices as part of IPV interventions in different settings and patient populations [28, 29]. The results show that women find digital tools feasible as part of violence interventions [28, 29] and that women are more likely to disclose IPV when digital tools are used for screening [30].

Mainstream interventions usually target the majority population and often fail to reach minority groups [31]. Interventions that are available in a woman’s mother tongue and involve experts from the target groups in the design of the studies support higher recruitment and programme utilisation [32]. Culturally sensitive interventions may result in more successful outcomes [33]. Few interventions that address IPV against pregnant women focus on cultural sensitivity [34].

To the best of our knowledge, no cultural sensitivity interventions using mHealth to address IPV amongst pregnant women have been conducted. We addressed this gap through the Safe Pregnancy trial, a tablet-based intervention in antenatal care that included information about violence and its consequences, education about safety behaviours and encouragement to talk to the midwife about violence. We hypothesised that the Safe Pregnancy intervention would prevent IPV and positively affect women’s health and quality of life through increased awareness and use of safety and help-seeking behaviours.

## Methods

### Study design

The Safe Pregnancy study was designed as a two-group, randomised controlled trial (RCT) conducted in routine antenatal care settings at 19 maternal and child health centres (MCHCs) in South-Eastern Norway [35]. We compared the effects of a culturally sensitive video that communicated information about violence and safety behaviours (intervention) and a video that communicated general information about lifestyle promoting a safe pregnancy (control) on abused women’s quality of life (WHOQOL-BREF) approximately three months post-partum. The trial was registered in clinicaltrials.gov on 11/01/2018, registration number NCT03397277, and is conformed to the CONSORT guidelines [36].

### Participants

**Fig. 1:**
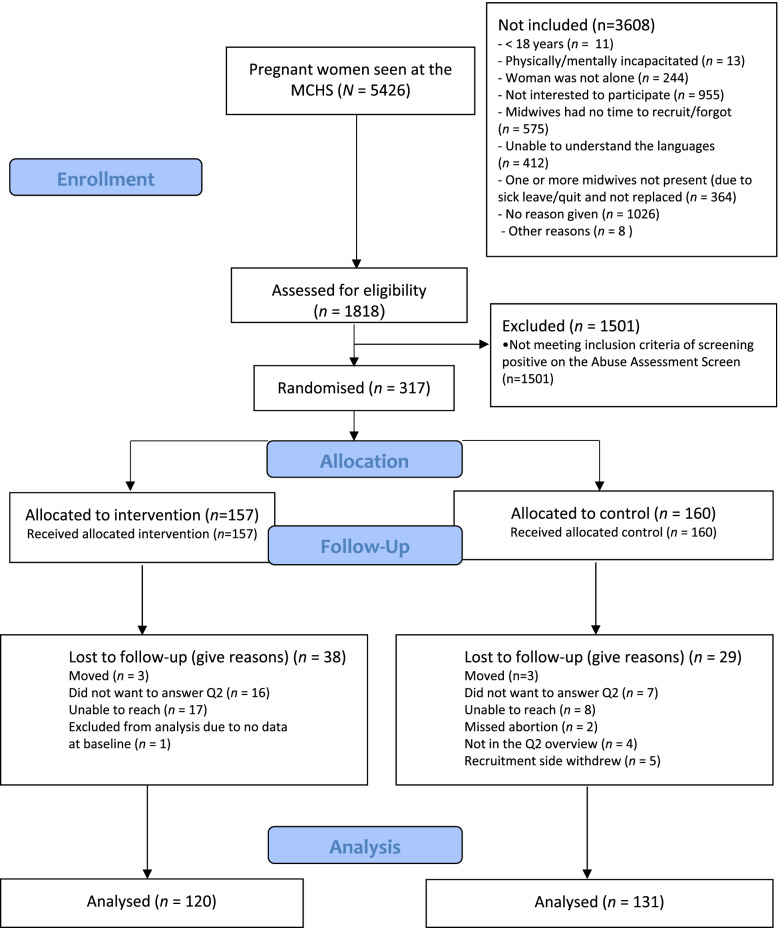
CONSORT 2010 flow diagram

Pregnant women aged 18 and above, at any gestational age and attending routine antenatal check-ups without their partner or other family members, were screened for previous and/or recent IPV experiences [35]. The participants were recruited at any time throughout the pregnancy. Women who had insufficient Norwegian, English, Urdu or Somali language skills and/or had difficulties responding because of cognitive impairment were excluded [35]. In all, 1818 women were assessed for eligibility, and 317 women who had experienced any lifetime IPV participated (Fig. 1).

### Recruitment and data collection

Prior to inclusion of the participants, a programme for the professional development of midwives at all MCHCs was conducted [35], which consisted of a conference addressing IPV during pregnancy, information about the trial, detailed instructions for the recruitment of participants and individual teaching sessions in use of a tablet. All midwives received a copy of the recruitment procedure. During the recruitment period, project meetings with a mix of presentations about different resources within the field and reflections were held.

The recruiting midwives introduced the study as one investigating factors associated with a safe pregnancy, including stress, quality of life and IPV during pregnancy. This strategy was to emphasise the focus on staying safe and healthy during pregnancy, thus masking the intervention and that it was about IPV to the women. Additionally, the participants received written information about the study, and the midwives obtained written consent from all participants.

The data were collected at the MCHCs using self-administered questionnaires on a tablet at baseline (Q1) and approximately three months post-partum (Q2). The women were given privacy to complete the questionnaires and could use headphones if they preferred. Most women answered Q2 at the MCHCs at a routine visit to the vaccination programme for the child. If they were short of time at this appointment, they were contacted by mobile phone by members of the project group. The project member asked whether she was by herself, and if so, sent the questionnaire link via SMS or e-mail. Recruitment period was from January 2018 to July 2019. Follow-up data collection was completed in June 2020. The women who declined to participate were given the opportunity to give their reasons: common reasons were that women had insufficient language skills, or they were not interested (Fig. 1). In addition, midwives sometimes forgot or were too busy to recruit (see Fig. 1).

### Ethical considerations

The Regional Committee for Medical and Health Research Ethics approved the study (ref. no: 2017/358). Additionally, the trial followed the Helsinki Protocol [37] and ethical and safety recommendations for research on IPV [38]. All MCHCs received an overview of services for women and families exposed to IPV, including referrals to community services, such as legal aid, police, child welfare services and crisis shelters. The participating women, irrespective of IPV disclosure, received an appointment card featuring a list of phone numbers and websites for government and local resources promoting safe pregnancy, as well as phone numbers for police and pre-hospital services. Additionally, both videos (intervention and control) encouraged women to speak to their midwife if they did not feel safe. The data were anonymised before analysis.

### Randomisation and blinding

Randomisation was performed on the tablet on a 1:1 basis with allocated blocks of 4. After completing Q1, all women who screened positively for violence were randomised to watch the intervention video or the control video. They were not informed about which video was the intervention or control. The women who screened negative for violence saw the control video. The researchers who performed the outcome analysis of the trial were blinded to the allocation of participants until after the analysis.

### Intervention, control and usual care

The intervention video used digital storytelling and consisted of images, pictures, sound and a video, focusing on information about the definition and types of IPV, the cycle of abuse, IPV during pregnancy and health consequences, help-seeking strategies and safety-promoting behaviours. Digital storytelling is a strategy to empower people and facilitate learning [39]. The video lasted approximately seven minutes and was provided in Norwegian, English, Urdu and Somali. The video contained pictures and images of women with diverse ethnic and cultural backgrounds [40].

The control video consisted of general information about different aspects of having a healthy and safe pregnancy, recommendations regarding a healthy diet, exercise, alcohol consumption and smoking and brief information about where to get help if exposed to IPV [35]. The control video also lasted approximately seven minutes.

All women received usual care based on national guidelines for antenatal care that instruct health professionals to routinely ask all women about their experiences of violence and provide assistance from community/municipality resources if necessary [17].

### Instruments

The World Health Organization Quality of Life-BREF (WHOQOL-BREF) instrument has been validated in multiple countries, including Norway, and has shown good cross-cultural validity, reliability and sensitivity as reflected by its four domains: physical, psychological, social and environmental [41]. The five-item Abuse Assessment Screen (AAS) was created to detect current or former abuse perpetrated against pregnant women. The instrument has been tested in an obstetric and gynaecological outpatient population in a face-to face setting in the U.S, Brazil and Sri Lanka and has shown good sensitivity (94%) and fair to good specificity (55–99%) [42]. The Composite Abuse Scale Short Form-Revised (CAS_R_-SF ) is a multidimensional instrument created to assess lifetime, recent (within the last 12 months) and current exposure and frequency of partner abuse. International IPV experts including academic researchers, service providers and policy actors, and pooled data from 6278 adult, English-speaking women collected in five Canadian studies, were included in the development of the CAS SF-R [43]. The instrument has demonstrated initial reliability and validity [43].

The use and adoption of safety behaviours was based on a 15-item checklist developed and tested by McFarlane et al. [44]. Detailed descriptions of the instruments have been published in the trial protocol [35] and the statistical analysis plan (Supplementary File).

All project material was professionally translated into Norwegian, English, Urdu and Somali. Norwegian, Pakistani and Somali women, with and without experiences of IPV, and professionals working at crisis shelters were involved in a qualitative user involvement study during the development of the intervention (questionnaire and video) [40]; the content, functionality, cultural sensitivity and feasibility were explored, and the participants provided important feedback, resulting in revisions [40].

### Background variables

The women reported sociodemographic, socio-economic and obstetric status by selecting predefined categories. For analytical purposes, the predefined categories were merged, as shown in Table 1. At baseline, women were asked how many weeks pregnant (gestational age) they were when completed the questionnaire, how many children they had given birth to (parity), their civil status, education level, employment status, joint family income, negative experiences related to alcohol consumption within the last year, including those of her partner and smoking during the pregnancy. We categorised women into native and non-native Norwegian speakers, using mother tongue as a determining factor, since this can be a true indicator of understanding and orienting oneself in a different culture [45].At follow-up, women reported how many weeks that had passed since they gave birth. The 19 MCHCs were categorised according to the number of women seen within a year; small (< 100), medium (100–300) and large (> 300) (Supplementary Table 1). One MCHC was recruited only for a short period and thus had no follow-up cases.

**Table 1:** Sociodemographic and obstetric characteristics of the total sample at baseline (*N* = 251)

	**Total**	**Intervention group** ***n* = 120**	**Control group *n* = 131**	***P-*value**
	***N* (%)**	**Mean (SD)**	**Mean (SD)**	
**Age**	251 (100)	31.33 (4.67)	31.69 (4.84)	0.557
**Gestational age**	251 (100)	27.83 (6.48)	29.76 (7.04)	0.024
		**Median (min–max)**	**Median (min–max)**	
**Weeks after birth at follow-up**	251 (100)	15 (6–58)	16 (6–56)	0.847
	***N* (%)**	***n* (%)**	***n* (%)**	
**Civil status**				0.836
Married/cohabiting	224 (89.2)	106 (88.3)	118 (90.1)	
Single	16 (6.4)	8 (6.7)	8 (6.1)	
Missing	11 (4.4)	6 (5.0)	5 (3.8)	
**Level of education**				0.873
≤ High school	89 (35.5)	44 (36.7)	45 (34.4)	
College/university < 4 years	77 (30.6)	35 (29.2)	42 (32.1)	
College/university ≥ 4 years	83 (33.1)	40 (33.3)	43 (32.7)	
Missing	2 (0.8)	1 (0.8)	1 (0.8)	
**Employment status**				0.325
Working/studying	193 (76.9)	89 (74.2)	104 (79.4)	
Unemployed	56 (22.3)	30 (25)	26 (19.8)	
Missing	2 (0.8)	1 (0.8)	1 (0.8)	
**Joint family income last year**				0.650
≤ NOK 599,000	57 (22.7)	31 (25.8)	26 (19.8)	
NOK 600–999,000	109 (43.4)	52 (43.3)	57 (43.5)	
≥ NOK 1,000,000	60 (23.9)	26 (21.7)	34 (26)	
Do not know	23 (9.2)	10 (8.4)	13 (9.9)	
Missing	2 (0.8)	1 (0.8)	1 (0.8)	
**Mother tongue**				0.370
Norwegian	190 (75.7)	88 (73.4)	102 (77.8)	
Somali	3 (1.2)	0	3 (2.3)	
Urdu	4 (1.6)	2 (1.7)	2 (1.5)	
English	2 (0.8)	1 (0.8)	1 (0.8)	
Other	50 (19.9)	28 (23.3)	22 (16.8)	
Missing	2 (0.8)	1 (0.8)	1 (0.8)	
**Parity**				0.280
Nulliparous	124 (49.4)	55 (45.8)	69 (52.6)	
Multiparous	125 (49.8)	64 (53.4)	61 (46.6)	
Missing	2 (0.8)	1 (0.8)	1 (0.8)	
**Tobacco use**				0.454
Yes	12 (4.8)	7 (5.8)	5 (3.8)	
No	239 (95.2)	113 (94.2)	126 (96.2)	
**Negative experiences with alcohol consumption (woman)**				0.381
Yes	34 (13.5)	14 (11.7)	20 (15.3)	
No	217 (82.5)	102 (85)	105 (80.1)	
Missing	10 (4)	4 (3.3)	6 (4.6)	
**Negative experiences with alcohol consumption (partner)**				0.996
Yes	36 (14.3)	17 (14.2)	19 (14.5)	
No	201 (80.1)	95 (79.1)	106 (80.9)	
Missing	14 (5.6)	8 (6.7)	6 (4.6)	
**Maternal and child health centre**				0.131
Small (< 100)	29 (11.6)	9 (7.5)	20 (15.3)	
Medium (100**–**300)	60 (23.9)	28 (23.3)	32 (24.4)	
Large (> 300)	162 (64.5)	83 (69.2)	79 (60.3)	

At baseline and follow-up, IPV exposure was measured by the AAS. The first question addresses fear of a partner or someone else. Questions two to five address different actions perpetrated by a current or former partner, representing fear, emotional, physical and sexual violence. The answer options were “never”, “yes, previously”, “yes, during the 12 months before pregnancy” and “yes, since the start of the pregnancy.” The responses were classified as “no IPV”, “previous IPV”, “recent IPV (during the 12 months before the pregnancy and during the pregnancy)” and “both previous and recent IPV”. The first question, addressing fear of a partner or someone else, was categorised as “fear”. The women who reported that their partner/ex-partner had done something to make them afraid of them were categorised as “afraid of partner”. The women who responded positively to the questions addressing emotional, physical and/or sexual violence were classified as having experienced either emotional, physical or sexual IPV. A history of IPV was determined by a positive answer to at least one of the five questions. For the logistic regression analyses at follow-up, the AAS answer options were merged into a dichotomous variable categorised as “yes” and “no” to IPV experiences. The women who reported a history of IPV at baseline and follow-up were asked to fill out the CAS_R_-SF and the 15-item Safety Behaviour checklist.

### Outcomes

The primary outcome was women’s perceived quality of life (QOL) within the last two weeks measured by the WHOQOL-BREF, including four domains: physical, psychological, social and environmental [41]. Additionally, this instrument includes two global items on the overall perception of QOL and health examined separately. Each item was scored on a Likert scale ranging from 1 to 5. As described in the WHOQOL-BREF manual [46], the mean score of the items within each domain is transformed linearly to a domain score scaled in a positive direction from 1 to 100. Higher scores are associated with higher QOL [46].

The secondary outcomes were the use of safety-promoting behaviours and exposure to recent IPV. The women considered a list of 15 safety behaviours at baseline and follow-up [47]. The answer options were “yes”, “no” and “not applicable”. We computed a sum score of used safety behaviours for all women by using the formula x = 15 * (a/b) where a/b was the proportion of used safety behaviours out of the number of applicable behaviours as all items on the list were not applicable to each woman. The range of the adjusted number was 0 and 15. A high number of adjusted scores indicated a high number of used safety-promoting behaviours.

Experiences of IPV within the last 12 months and/or during pregnancy were measured by the CAS_R_-SF, including 15 descriptive questions that captured the frequency (0–5 scale) and the severity of different actions of emotional, physical, sexual and overall IPV. The total score ranges from 0 to 75, with higher scores associated with greater exposure to IPV. For the questionnaire to be valid, no more than 4 items (out of 15) could be missing [43].

### Changes to the protocol

Initially, we had two main outcomes: the use of safety behaviours and participants’ QOL. We changed this to QOL as the main outcome with use of safety behaviours as a secondary outcome. We planned to perform stratified analyses for women whose mother tongue was Norwegian, English, Urdu, Somali or other. However, our study had insufficient power to perform the intended sub-analyses. Nevertheless, we examined the differences between the native Norwegian speakers and the non-native Norwegian speakers.

### Sample size

We hypothesised that there would be no change in QOL in the control group and approximately a 5% change in the intervention group. Assuming that the QOL at baseline was about 80 [48], a 5% change would be 4 points. To reveal such a change as statistically significant, keeping the power to 80% and the significance level alpha to 5%, we would need 100 participants in each group. As we included 120 and 131 in the intervention and the control group, respectively, we consider our study to be sufficiently powered.

### Statistical analyses

The baseline characteristics are presented as counts and percentages for the categorical variables and means and standard deviations for the continuous variables. The differences in the sociodemographic, obstetric and IPV variables were analysed using the Pearson chi-square test for the categorical data and the independent sample t-test for the continuous variables when the data were normally distributed. The Mann–Whitney-Wilcoxon test was used when the data were skewed. To assess baseline representativeness, the differences in characteristics between the responders and those lost to follow-up were examined.

Differences in perceived QOL, the use of safety behaviours and the CAS SF-R scores were estimated using the general linear model for repeated measures. We included all the subjects with baseline and follow-up data without imputing missing values. All analyses were performed according to intention to treat principals. The covariates, gestational age and access to videos, were entered as fixed effects. Access to videos versus time of analysis interactions were modelled. The MCHCs were entered as a random effect. The results are presented as estimated means with 95% confidence intervals (CIs). Fixed effects are presented as *P*-values. Comparisons were performed between and within the groups.

To examine the association between a history of IPV (AAS) and background variables at follow-up, logistic regression analyses were performed including subjects with baseline and follow-up data. Variables with a *P*-value < 0.1 in the univariate analyses were included in the multivariate analysis: education, access to videos, weeks since giving birth, civil status and employment status.

*P*-values < 0.05 were considered as statistically significant. All analyses were conducted using IBM SPSS Statistics 27 (IBM Corp., Armonk, NY, USA).

## Results

At baseline, 1818 women were assessed for eligibility, and 317 (17.4%) reported a history of IPV and thus were randomised to the intervention (*n* = 157 [49.5%]) or the control group (*n* = 160 [50.5%]) (Table 1). A total of 66 (20.8%) women were lost to follow-up. Data from 251 women, 120 (76.4%) in the intervention group and 131 (81.9%) in the control group, were included in the analysis (see Fig. 1). Of the 251 women with IPV experiences, 39 (15.5%) reported IPV experiences during the last 12 months (data not in table).

The intervention group and the control group had similar sociodemographic baseline characteristics (Table 1). Of all the women, the mean age was 31.5 years (SD 4.75 [range: 20–47]) (data not in table). The proportion of native Norwegian speakers was 75.7%, and 23.5% were non-native Norwegian speakers (English 0.8%, Urdu 1.6%, Somali 1.2% and other 19.9%) (Table 1). In this study, almost two thirds of the included women had pursued higher education (college or university) (Table 1).

There were no differences at baseline between the groups regarding proportion of women who reported a history of IPV. Fear of partner (62.6%) and emotional IPV (68.5%) were the most common forms of violence (Table 2). Most of the women in the intervention group and the control group reported previous experiences of being afraid of a partner (62.5% and 55%, respectively) and emotional IPV (67.5% and 61.8%, respectively), rather than recent experiences of being afraid of a partner (2.5% and 4.6%, respectively) or emotional IPV (2.5% and 2.3%, respectively)(Table 2).

**Table 2:** History of violence at baseline (*N* = 251)

AAS	Total	Intervention group *n* = 120	Control group *n* = 131	*P-*value
	***N*** (**%)**	***n* (%)**	***n* (%)**	
**Fear**				0.139
No	99 (39.4)	51 (42.5)	48 (36.6)	
Previous	119 (47.4)	57 (47.5)	62 (47.3)	
Recent	23 (9.2)	6 (5.0)	17 (13.0)	
Previous and recent	10 (4.0)	6 (5.0)	4 (3.1)	
**Afraid of partner**				0.346
No	93 (37)	40 (33.4)	53 (40.4)	
Previous	147 (58.6)	75 (62.5)	72 (55.0)	
Recent	9 (3.6)	3 (2.5)	6 (4.6)	
Previous and recent	1 (0.4)	1 (0.8)	0 (0)	
Missing	1 (0.4)	1 (0.8)		
**Emotional IPV**				0.484
No	78 (31.1)	33 (27.5)	45 (34.3)	
Previously	162 (64.5)	81 (67.5)	81 (61.8)	
Recent	6 (2.4)	3 (2.5)	3 (2.3)	
Previous and recent	4 (1.6)	3 (2.5)	1 (0.8)	
Missing	1 (0.4)		1 (0.8)	
**Physical IPV**				0.213
No	172 (68.5)	85 (70.9)	87 (66.4)	
Previous	74 (29.5)	31 (25.8)	43 (32.8)	
Recent	2 (0.8)	1 (0.8)	1 (0.8)	
Previous and recent	3 (1.2)	3 (2.5)	0 (0)	
**Sexual IPV**				0.194
No	197 (78.4)	89 (74.2)	108 (82.4)	
Previous	53 (21.2)	30 (25.0)	23 (17.6)	
Recent	1 (0.4)	1 (0.8)	0 (0)	
Previous and recent	0 (0)	0 (0)	0 (0)	

The analyses that compared the responders and women lost to follow-up showed no significant differences between the groups regarding sociodemographic background characteristics, thus indicating no selection bias (Supplementary Table 1). However, the women who were lost to follow-up were more likely to report recent emotional IPV and physical IPV (7.6% and 3%, respectively) compared to those who did respond (2.4% and 0.8%, respectively) (Supplementary Table 2) and less likely to report previous exposure to sexual IPV compared to those who did respond (4.5% and 21.1%, respectively) (Supplementary Table 2).

Assessing our main outcome, quality of life scores, we detected no significant score differences between the groups in any of the QOL domains, including overall perception of health and QOL, from baseline to follow-up (Table 3). Both groups showed an increase in physical health and a decrease in social relationships within the groups from baseline to follow-up. However, these differences were not statistically significant (Table 3).

**Table 3:** Primary outcome: quality of life (*N* = 251)

	**Study group**		***P*-value n**
	**Intervention group**	**Control group**	
**Time**	Estimated mean (95% CI)	Estimated mean (95% CI)	
**Overall QOL**			0.938
**Baseline**	4.24 (4.11–4.37)	4.22 (4.10–4.34)	
**3 months**	4.34 (4.21–4.46)	4.32 (4.20–4.44)	
**Overall health**			0.160
**Baseline**	3.87 (3.72–4.02)	3.85 (3.70–3.99)	
**3 months**	3.92 (3.77–4.07)	3.74 (3.59–3.88)	
**Physical health domain**			0.374
**Baseline**	49.92 (47.81–52.03)	48.42 (46.40–50.44)	
**3 months**	51.69 (49.56–53.81)	51.63 (49.58–53.67)	
**Psychological domain**			0.373
**Baseline**	67.33 (65.48–69.17)	67.24 (65.50–69.02)	
**3 months**	67.60 (65.76–69.43)	68.63 (66.86–70.40)	
**Social relationships domain**			0.930
**Baseline**	69.96 (66.85–73.08)	70.59 (67.60–73.58)	
**3 months**	67.69 (64.55–70.83)	68.13 (65.09–71.66)	
**Environmental domain**			0.097
**Baseline**	76.82 (74.58–79.06)	76.57 (74.43–78.70)	
**3 months**	76.96 (74.83–79.10)	78.87 (76.81–80.93)	

Covariates: gestational age, videos: intervention and control. Random effect: maternal and child health centre.

The use of safety behaviours increased between baseline and follow-up within both groups. However, no differences between the groups were observed (Table 4). We detected no differences in the frequency and severity of recent IPV between the groups from baseline to follow-up (Table 4). However, the women in the intervention group reported a small increase in mean overall IPV and emotional IPV scores, and they were more likely to report a decrease in the level of physical and sexual IPV, in contrast to women in the control group, who reported a decrease in mean overall IPV and emotional IPV scores (Table 4).

**Table 4:** Secondary outcomes: Use of safety behaviours and Composite Abuse Scale (CAS_R_-SF)

**Secondary outcome**	**Study group**		***P*-value n**
	**Intervention group**	**Control group**	
	Estimated mean (95% CI)	Estimated mean (95% CI)	
**Adjusted use of safety behaviours (*n* = 221)**			0.922
**Baseline**	1.86 (1.31–2.41)	1.38 (0.85–1.91)	
**3 months**	2.08 (0.96–3.20)	1.51 (0.32–2.70)	
**CAS**_**R**_**-SF**			
**Overall IPV** **(*n* = 153)**			0.156
**Baseline**	10.70 (7.24–14.16)	12.75 (9.18–16.33)	
**3 months**	11.17 (7.05–15.29)	8.54 (3.42–13.68)	
**Physical IPV** **(*n* = 74)**			0.191
**Baseline**	6.40 (3.06–9.74)	3.03 (-0.53–6.58)	
**3 months**	2.17 (0.11–4.22)	2.37 (0.03–4.70)	
**Emotional IPV** **(*n* = 139)**			0.106
**Baseline**	11.50 (7.40–15.59)	14.37 (10.26–18.47)	
**3 months**	12.48 (7.82–17.14)	9.21 (3.50–14.92)	
**Sexual IPV (*n* = 47)**			0.474
**Baseline**	6.70 (0.85–12.55)	5.07 (-1.23–11.37)	
**3 months**	2.24 (-2.81–7.29)	4.53 (-1.77–10.83)	

Covariates: gestational age, videos: intervention and control. Random effect: maternal and child health centre.

At follow-up, 115 (45.8%) women reported a history of IPV, while 136 (54.2%) did not (data not in table). Therefore, we examined the association between a history of violence and background characteristics at follow-up (Table 5). The control group women were less likely to report IPV than the intervention group women (odds ratio [OR] = 0.64 [95% CI 0.39–1.05]), borderline significant, *P* = 0.076). The association was attenuated and no longer significant when adjusting for sociodemographic covariates (weeks since birth, civil status, employment status and education level).

**Table 5:** Crude odds ratios and adjusted odds ratios for history of violence at follow-up (AAS) (*N* = 251)

Variables	Univariate			Multivariate		
	**OR**	**95% CI**	***P-*value**	**OR**	**95% CI**	***P-*value**
**Weeks since birth**	0.98	(0.95–1.01)	0.107	0.98	(0.95–1.01)	0.188
**Video**						
Intervention		ref			ref	
Control	0.64	(0.39–1.05)	0.076	0.65	(0.38–1.09)	0.101
**Civil status**						
Married/cohabitant		ref			ref	
Single	2.68	(0.90–7.96)	0.076	2.12	(0.68–6.59)	0.194
**Employment status**						
Working/studying		ref			ref	
Unemployed	1.77	(0.97–3.22)	0.062	1.58	(0.84–2.97)	0.153
**Education**						
≤ High school	1.88	(1.02–3.52)	0.043	1.73	(0.90–3.02)	0.100
College or university < 4 years	1.55	(0.83–2.92)	0.172	1.56	(0.80–3.02)	0.191
College or university ≥ 4 years		ref			ref	

Covariates: weeks since birth, videos: intervention and control, civil status, employment status and education.

## Discussion

In our trial, a tablet-based intervention with a video containing information about IPV and safety behaviours that was compared to a video with general information about lifestyle promoting a safe pregnancy did not improve women’s QOL, use of safety behaviours or exposure to violence. Most participating women reported previous exposure to violence, and only a few women said they were exposed to violence during pregnancy. Approximately half of the women did not report previous violence exposure when asked again at the three-month follow-up.

We did not find that the women who received the IPV intervention reported improved QOL three months post-partum. This is supported by the findings of some studies [49–51] but is in contrast to the study by Tiwari and colleagues, who found that pregnant women who received a short face-to-face empowerment intervention reported significantly improved health-related QOL six weeks post-partum [26]. However, Tiwari and colleagues used a different intervention and another instrument to measure QOL in their study [26]. In addition, they used the original AAS and included women who were exposed to violence in the 12 months before pregnancy and during pregnancy. Our decision on including women with past exposure to violence may have diluted the effect of the intervention. Research has shown that IPV is associated with poorer QOL during pregnancy [52] and that both previous and recent IPV experiences are associated with long-term poorer QOL [53]. Hence, measuring QOL is relevant but may require longer follow-up than three months post-partum.

A few women in our study used the safety behaviours facilitated in the intervention video. Studies have assessed changes in safety-promoting behaviours, with conflicting results [54]. The original safety behaviour list was implemented by McFarlane et al. [55]. An RCT conducted in primary care amongst women exposed to violence within the last 12 months, which compared abuse assessment and a referral card with abuse assessment and a 20-min nurse case management protocol, detected no differences between the groups [47]. However, the safety-promoting behaviours increased over time in both groups [47]. The mean number of safety behaviours used was approximately 10 in both groups, compared to our study, with a mean number of used safety behaviours of between 1 and 2. In contrast to McFarlane et al., few women in our study reported ongoing violence; thus, the safety behaviours may not have been applicable for the women in our study.

We measured ongoing violence using the CAS_R_-SF, which includes 15 descriptive questions about the frequency and severity of emotional, physical, sexual and overall IPV [43]. Few women reported violence during the last 12 months, including the current pregnancy. Emotional IPV was the most common form of violence. We did not detect any differences in violence exposure between the intervention group and the control group at three months post-partum. This finding is supported by other studies [51, 56–58]. Few studies that offered different types of supportive counselling or short interventions during pregnancy have shown a statistically significant decrease in various forms of violence [9]. There are challenges associated with research addressing violence, and several ethical and safety issues need to be taken into consideration [9]. Most studies ask both the intervention and the control groups about violence exposure at baseline and offer some information about IPV referrals or resources to the control group [9]. This may be more effective than anticipated and dilute the intervention’s effect.

Surprisingly, less than half of the women who screened positive for IPV on the modified AAS at baseline screened negative at follow-up. The AAS was created to detect violence against pregnant women [59]. It is shown to have good sensitivity (over 93–94%) and fair to good specificity (from 55–99%) when used by trained clinicians in a face-to-face setting [42]. In our study, we had a different setting using a tablet, but this was used at both baseline and follow up. Hence, the data collection was similar at both timepoints, and a similar prevalence of violence was expected.. To our knowledge, the AAS has not been validated in a Norwegian pregnant population and we did not validate the modified version that we used. However, we performed a user involvement study when we developed the tools in this intervention, and the women expressed no difficulties related to the AAS questions and the modifications made were mostly based on their input [40].

All the women who answered “yes” to one or more of the five AAS questions at baseline were included in this RCT. The first two questions, addressing fear of partner or someone else or if the partner/ex-partner had done anything to make the woman afraid, may have been too unspecific, hence the different answer at follow-up. Alternatively, since the majority reported previous experiences, the women may have thought differently about the situation when asked again after their child’s birth. When we examined the factors associated with reporting violence at follow-up, we found that being in the intervention group was associated with reporting violence again. This may be due to the information about IPV in the intervention video and more women realising that they had been exposed to IPV. However, the association was attenuated and no longer significant when we controlled for sociodemographic variables.

Approximately one in five of the women was lost to follow-up three months post-partum in this study. The baseline characteristics of the responders and the non-responders did not differ. However, the women in the intervention group who were lost to follow-up tended to report recent emotional IPV more frequently compared to the control group women who were lost to follow-up. The likelihood of reporting ongoing violence or more severe forms of violence amongst women lost to follow-up is common in IPV studies [25, 60].

We used tablet-based technology to test a short intervention in this study and did not detect any differences between the intervention and the control groups. mHealth technology has been used in IPV interventions, mainly as a screening tool [29, 49], suggesting that this yields higher detection rates [30], or as a method to facilitate discussion about IPV [29]. No other studies in antenatal care have delivered the intervention itself using mHealth; hence, it is difficult to compare our findings to others. A study by Koziol-McLain et al. that tested a short intervention similar to ours, but face-to-face in an emergency room setting, did not find that the intervention reduced violence or enabled more use of safety behaviours at three months’ follow-up [61]. There is insufficient evidence of effective interventions for IPV during pregnancy [9, 18]. The most promising results from interventions in the antenatal period are shown in multifaceted interventions, such as home visitation programmes and mentor support [25, 62], that is, more complex interventions than a short intervention video.

The present trial included culturally diverse pregnant women living in South-Eastern Norway. Even though the non-native Norwegian-speaking women may originate from other high-income countries, as well as low- and middle-income countries, they may still have a linguistic and cultural barrier when they communicate with Norwegian health professionals about sensitive topics. Although we tried to tailor the intervention specifically to women with Pakistani and Somali backgrounds, the availability of the intervention in Urdu and Somali as well as the user involvement study [40] was not enough to recruit more women from our target groups. Communication about sensitive topics seems to be especially challenging when health professionals serve a multicultural population, which reveals the need for culturally sensitive communication strategies [63].

The strengths of the present study were the large number of MCHCs and thus the large number of participants, including a high number of non-native Norwegian speakers. The RCT design facilitated methodological qualities such as computerised randomisation and a blinded intervention that minimised performance and selection bias and blinded the analyses of the outcomes. Additionally, we used several well-validated instruments [41–43].

A further strength of the trial is that it was adequately powered; thus, the external validity is satisfactory. However, a significant limitation is that few women reported violence during pregnancy, the primary target group for our intervention. Thus, the results should be interpreted with caution. It is not unlikely that the intervention would have been effectful if we had reached the intended group. Women may not always disclose the true nature of IPV, and its prevalence may be underreported [3]. Ideally, we would have asked the women about IPV exposure several times during pregnancy since this may increase the disclosure rate [64].

Another limitation is that we cannot know for sure that the women watched the videos because they were alone while answering the questionnaire. In addition, the women were offered the tablet-based intervention only once during the study. If the intervention had been offered several times during antenatal care, it may have had a positive effect. The three-month follow-up in our study may have been too short to detect any difference between the groups. The DOVE study by Sharps et al., an IPV enhanced home visitation program intervened several times during the antenatal period with a long time follow-up, provided evidence of long-term decreased IPV [24].

A total of, 63.7% of our sample reported higher educational levels, whereas the average in the Norwegian female population is 37% [65], and the result should therefore be interpreted with caution in pregnant populations that report educational levels according to the average of Norwegian women.

Although we did not detect any effect at the three-month follow-up, our tablet-based instrument can still be used in antenatal care. In qualitative interviews with women and midwives participating in the Safe Pregnancy trial, both groups viewed it as a supplement to face-to-face communication [66, 67]. Further, the women suggested making the tablet intervention available in other settings where women meet health care professionals [66]. The midwives reported that the short intervention video made it easier to address IPV, and as a help in a time-limited setting with many tasks and demands [67]. There is evidence that asking directly via an mHealth device if the user needs help can be valuable [68]. The possibility to ask directly can be added as a tool to our tablet-based intervention. The intervention used in this study can also be feasibly replicated in other languages.

## Conclusions

Our findings reveal the need for further research into and development of tablet-based interventions for women experiencing IPV, particularly culturally sensitive interventions for women with a multicultural background. The tablet-based intervention should be directed towards women who are or have been in a recent abusive relationship. In addition, our instrument may be a useful tool to facilitate motivation for women experiencing IPV to seek help and support from the midwife. It may also generate a query for midwives to ask directly about IPV experiences.

## Supplementary Information

**Additional file 1. **

**Additional file 2. **

## Data Availability

The datasets used and/or analysed during the current study are available from the corresponding author on reasonable request.

## Abbreviations

AAS: Abuse Assessment Screen
CAS_R_-SF: Composite Abuse Scale Short Form – Revised
IPV: Intimate partner violence
MCHC: Maternal and child health centre
mHealth: Mobile health
Q1: Questionnaire one;
Q2: Questionnaire two
QOL: Quality of life
RCT: Randomised controlled trial
WHOQOL-BREF: World Health Organization Quality of Life –BREF
