# Supplementary material for: Safe Pregnancy intervention for intimate partner violence: a randomised controlled trial in Norway among culturally diverse pregnant women
Source: BMC Pregnancy Childbirth. 2022 Feb 21;22:144. doi: 10.1186/s12884-022-04400-z (PMC8862262; doi:10.1186/s12884-022-04400-z)

**Preparing for the worst – promoting safety behaviours in antenatal care among Norwegian, Pakistani and Somali pregnant women. A randomised controlled trial**

**Statistical Analysis Plan 18^th^ December 2020**

**Authors:**

Eva Marie Engebakken Flaathen*

Lena Henriksen*

Milada Småstuen

Berit Schei

Angela Taft

Josef Noll

Lisa Garnweidner-Holme

Mirjam Lukasse

*Joint first authorship

Table of Contents

[Study synopsis 3](#_Toc88128945)

[Objectives 3](#_Toc88128946)

[Design 3](#_Toc88128947)

[Participants 3](#_Toc88128948)

[Procedures 3](#_Toc88128949)

[Outcomes 3](#_Toc88128950)

[Statistical analyses 5](#_Toc88128951)

[Sample size 6](#_Toc88128952)

[Procedures for analysis 6](#_Toc88128953)

[Description of study flow and study sample 6](#_Toc88128954)

[Description of missing data 6](#_Toc88128955)

[Outcome 6](#_Toc88128956)

[Tables 7](#_Toc88128957)

[References 13](#_Toc88128958)

# Study synopsis

## Objectives

The main objective of this study is to measure the effect of a culturally sensitive intervention promoting safety behaviors to prevent and reduce IPV among Norwegian, Pakistani and Somali pregnant women experiencing intimate partner violence.

## Design

The study is a randomised controlled trial (RCT) conducted in 19 mother and child centres (MCHC) located in South-Eastern Norway. The recruitment of participants took place from January 2018 to July 2019, with follow up data collection finishing in June 2020. We will compare women`s perception of quality of life in two groups post-partum; the group who received the intervention video and the group who received the control video (1).

We will follow the recommendations for reporting RCTs: Randomized (and quasi-randomized) controlled trial - CONSORT - Consolidated Standards of Reporting Trials http://www.equator-network.org/reporting-guidelines/consort/

## Participants

At baseline (Q1), 317 women were randomized to either the intervention video or the control video. A total of 251 women completed the follow up questionnaire (Q2) approximately 3 months post-partum and are included in the analyses.

## Procedures

Data is based on self-reporting questionnaires at baseline (any time during the pregnancy) and approximately 3 months post-partum.

## Outcomes

The primary outcome is:

1. **World Health Organization Quality of Life – Bref (WHOQOL-BREF):** Quality of life has been measured with the WHOQOL-BREF. (2). It consists of two global items on overall quality of life and general health, and four domains: Physical health domain (7 items), Psychological domain (6 items), Social relationships domain (3 items), and Environmental domain (8 items). This generates a profile of domain scores. The two additional items will be examined separately: the overall perception of quality of life and overall perception of health. Each item is scored on a Likert scale ranging from 1 to 5. The items ask the respondent "how much," "how often," "how completely," "how good" or "how satisfied" she felt about different aspects of her life in the past 2 weeks. The mean score of the items within each domain is transformed linearly to a domain score scaled in a positive direction from 0–100, such that higher scores indicate higher quality of life.

The secondary outcomes are:

1. **Use of safety behaviours:** The list of 15 safety behaviours was developed by Mc Farlane et al 2002 (3) and 2004 (4). The list is adapted to a Norwegian setting. It still consists of 15 safety behaviours that women have considered. The answering options were yes, no, not applicable. We will compute the sum score and adjust for number of not applicable answers as follows:

x= 15 * (a/b) where a/b is the proportion of recognized safety behaviours out of the number of applicable behaviours. Thus, the adjusted total falls between 0 and 15. The equation used to calculate the adjusted total is: *a*/*b*= *x*/15, where a is the number of behaviours performed, *b* is the number of behaviours applicable, and x is the adjusted total. When *a* and *b* are known the adjusted total number can be calculated by cross-multiplying the two fractions. Our hypothesis is that an increase in the numbers of safety promoting behaviours is positive.

1. **Composite Abuse Scale (CAS):** CAS R-SF is a 15-item instrument that captures physical, sexual and psychological abuse and overall Intimate Partner violence (IPV) (5). Women have answered 15 questions about different actions, and they had the possibility to answer: Has this ever happened to you? Yes/No. If yes, how often did it happened in the last 12 months: Not in the past 12 months, once, a few times, monthly, weekly, daily/almost daily (0 to 5 scale). Total scores for the CAS R-SF, ranging from 0 to 75, will be calculated by computing mean of past 12-month frequency of abuse and multiplying by 15. For the questionnaire to be valid, no more than 3 items (out of 15) can be missing. Subscale scores will be calculated for the physical, sexual and psychological abuse in a similar manner.

## Statistical analyses

The study is an RCT where two groups will be compared, the group who received the intervention video and the group who received the control video. Descriptive statistics will be used to present the characteristics of participants in the intervention group and the control group, providing frequencies (counts) and properties (percentages) for categorical variables. Continuous variables will be presented with means and standard deviations (SD).

Characteristics of the women in the two different groups will be compared to check if the groups are balanced concerning background variables and possible confounders. We expect the randomization to take care of a similar distribution of the characteristics of the women participating in relation to socio-economic factors such as age, education, ethnic background, economic status and experiences of IPV measured on the Abuse Assessment Scale (6). We also expect the randomization to create a similar distribution of medical and obstetric factors such as parity, gestational age and the time of filling out the questionnaire. However, we will check if our assumptions are correct and compare these using X^2^ test for categorical variables and independent T-test for continuous variables when the data are normally distributed. The Mann-Whitney-Wilcoxon test will be used if the data are skewed.

For the main and secondary outcome (continuous variables), we will fit linear multiple mixed effects models with the unit as a random effect, group as the fixed effect and selected

covariates (background variables if not similarly distributed between the groups) as possible confounders to assess the possible effect of the intervention. Further, we will perform sensitivity analyses to assess a possible effect of the Maternal and Child Health Centres (MCHC) on the different outcomes. If there are significant differences between the MCHCs, we will treat MCHC as a fixed covariate and thus adjust for possible confounding in the multiple model. If there is an interaction between a group (intervention vs control) and a MCHC, we will present stratified analyses. We will perform sensitivity analyses to explore different categorization of the MCHCs based on the number of women at each site.

According to the published protocol (1), we planned to perform stratified analyses for native Norwegian speakers and non-native Norwegian speakers as we anticipated differences between these groups. Our study has insufficient power to perform the intended sub-analyses for ethnicity. However, we will examine the differences between native Norwegian speakers and non-native Norwegian speakers (Somali 1.0 %, Pakistani 4.2 %, English 0.8 % and others 20.8 %).

Intention to treat analysis will be performed. All tests will be two-sided. *P*-values <0.05 will be considered statistically significant.

## Sample size

We anticipated no change in quality of life in the control group and about 5% change in the intervention group. Assuming the quality of life at baseline was about 80, a 5% change would be 4 points. To reveal such a change as statistically significant keeping power to 80% and significance level alpha to 5%, we would need 100 participants in each group.

Given we have 120 and 131 in each group, we consider our study sufficiently powered.

## Procedures for analysis

The analysis will be conducted by Eva Marie Engebakken Flaathen (EMEF), Lena Henriksen (LH) and Milada Småstuen (MS) using SPSS and STATA. EMEF and LH will conduct the descriptive analyses to present the characteristics of the intervention and control group (Table 1) and MS will be responsible for the rest of the analysis.

## Description of study flow and study sample

The flow of participants through the study will be reported according to the Consolidated Standards of Reporting Trials (CONSORT) flow diagram for individual randomized controlled trials of non-pharmacological treatments. Reasons for exclusion and loss to follow-up will be provided where known.

The study sample will be described in detail using data obtained at baseline. Table 1 shows the variables that will be used to describe the sample.

## Description of missing data

Due to small data model-based imputation of missing data will not be performed.

## Outcome

The primary outcome is quality of life among approximately 3 months post-partum. The secondary outcome is the adaption of the safety behaviors (3, 4) and experiences of physical, sexual, emotional and overall intimate partner violence during the past 12 months or during the pregnancy measured on the Composite Abused Scale (CAS R-SF) (5).

## Tables

**Table 1.** Socio-demographic characteristics at baseline in the Safe Pregnancy study, n=251.

| Characteristic |  | | The intervention group *n* = | | The control group *n* = | | *P-*value |
| --- | --- | --- | --- | --- | --- | --- | --- |
|  | *n* | (%) | *n* | (%) | *n* | (%) |  |
| Age |  |  |  |  |  |  |  |
| Missing |  |  |  |  |  |  |  |
| GA when filling out Q1 |  |  |  |  |  |  |  |
| Civil status |  |  |  |  |  |  |  |
| Married/living with partner |  |  |  |  |  |  |  |
| Other |  |  |  |  |  |  |  |
| Missing |  |  |  |  |  |  |  |
| Education |  |  |  |  |  |  |  |
| High school ≤ 13 years |  |  |  |  |  |  |  |
| College/university less than 4 years |  |  |  |  |  |  |  |
| College/university more than 4 years |  |  |  |  |  |  |  |
| Missing |  |  |  |  |  |  |  |
| Occupation |  |  |  |  |  |  |  |
| Employed or self-employed |  |  |  |  |  |  |  |
| Not employed |  |  |  |  |  |  |  |
| Missing |  |  |  |  |  |  |  |
| Joint family income last year |  |  |  |  |  |  |  |
| < 599.000 NOK |  |  |  |  |  |  |  |
| 600–999.000 NOK |  |  |  |  |  |  |  |
| > 1000.000 NOK |  |  |  |  |  |  |  |
| Do not know |  |  |  |  |  |  |  |
| Missing |  |  |  |  |  |  |  |
| Ethnicity |  |  |  |  |  |  |  |
| Norwegian |  |  |  |  |  |  |  |
| Other |  |  |  |  |  |  |  |
| Missing |  |  |  |  |  |  |  |
| Parity |  |  |  |  |  |  |  |
| P0 |  |  |  |  |  |  |  |
| ≥ P1 |  |  |  |  |  |  |  |
| Missing |  |  |  |  |  |  |  |
| IPV (AAS) |  |  |  |  |  |  |  |
| Fear  (Previous and recent) |  |  |  |  |  |  |  |
| Emotional IPV  (Previous and recent) |  |  |  |  |  |  |  |
| Physical IPV  (Previous and recent) |  |  |  |  |  |  |  |
| Sexual IPV  (Previous and recent) |  |  |  |  |  |  |  |
| Tobacco use |  | |  |  |  |  |  |
| Yes |  |  |  |  |  |  |  |
| Missing |  |  |  |  |  |  |  |
| Alcohol use |  | |  |  |  |  |  |
| Yes |  |  |  |  |  |  |  |
| Missing |  |  |  |  |  |  |  |

**Table 2.** Primary outcome: Quality of life (WHOQOL-Bref)

|  | Study group | | | Analysis | | p-value n | |
| --- | --- | --- | --- | --- | --- | --- | --- |
| Primary outcome | Intervention | | Control | Estimated effect size | |  | |
|  | N mean (SD) |  | |  | |  | |
| WHO Quality of life-BREF |  |  | |  | |  | |
| Overall QOL |  |  | | |  | |  |
| Baseline |  |  | | |  | |  |
| 3 months |  |  | | |  | |  |
| Physical health domain |  |  | |  | |  | |
| Baseline |  |  | |  | |  | |
| 3 months |  |  | |  | |  | |
| Psychological domain |  |  | |  | |  | |
| Baseline |  |  | |  | |  | |
| 3 months |  |  | |  | |  | |
| Social relationships domain |  |  | |  | |  | |
| Baseline |  |  | |  | |  | |
| 3 months |  |  | |  | |  | |
| Environmental domain | 0-100 |  | |  | |  | |
| Baseline |  |  | |  | |  | |
| 3 months |  |  | |  | |  | |

**Table 3.** Secondary outcome: Safety Behaviours and Composite Abuse Scale (CAS R-SF)

|  | Study group | | | Analysis | p-value n |
| --- | --- | --- | --- | --- | --- |
| Secondary outcome | The intervention group n= | | The control group n= |  |  |
|  | Estimated mean (95%C1) | Estimated mean (95%C1) | | Estimated between group difference (95% CI) |  |
| Safety behaviours |  |  | |  |  |
| Baseline |  |  | |  |  |
| 3 months |  |  | |  |  |
| Secondary outcome |  |  | |  |  |
| CAS R-SF |  |  | |  |  |
| Any IPV |  |  | |  |  |
| Baseline |  |  | |  |  |
| 3 months |  |  | |  |  |
| Emotional IPV |  |  | |  |  |
| Baseline |  |  | |  |  |
| 3 months |  |  | |  |  |
| Physical IPV |  |  | |  |  |
| Baseline |  |  | |  |  |
| 3 months |  |  | |  |  |
| Sexual IPV |  |  | |  |  |
| Baseline |  |  | |  |  |
| 3 months |  |  | |  |  |

**Table 4:** Mother and Child Health Centre/recruitment site

| Characteristic |  | | The intervention group *n* = | | The control group *n* = | | *P-*value |
| --- | --- | --- | --- | --- | --- | --- | --- |
|  | *n* | (%) | *n* | (%) | *n* | (%) |  |
|  |  | |  |  |  |  |  |
| Small MCHC |  |  |  |  |  |  |  |
| Medium MCHC |  |  |  |  |  |  |  |
| Large MCHC |  |  |  |  |  |  |  |

**Supplementary tables:**

**Table S1:** Percentage of women using each Safety Behaviours (SB) at baseline and 3 months post partum

| SAFETY BEHAVIORS | The intervention group *n* = | | The control group *n* = | | P value |
| --- | --- | --- | --- | --- | --- |
|  | Baseline | 3 months | Baseline | 3 months |  |
|  |  |  |  |  |  |
| Have you ever: |  | | | |  |
| …hid money? |  |  |  |  |  |
| … hid keys? |  |  |  |  |  |
| … established a code |  |  |  |  |  |
| … asked the neighbor to call police if violence begins? |  |  |  |  |  |
| … removed weapons (such as knives)? |  |  |  |  |  |
| … told someone how things are at home? |  |  |  |  |  |
| … stayed at a crisis shelter? |  |  |  |  |  |
| … documented bruises or violent events |  |  |  |  |  |
| Have you ever made sure you had available? | | | | |  |
| … social Security Numbers (yours, his, children)? |  |  |  |  |  |
| … passport/ID or other important papers |  |  |  |  |  |
| … your own bank account? |  |  |  |  |  |
| … valuable jewellery? |  |  |  |  |  |
| … a bag of extra clothing |  |  |  |  |  |
| … an extra phone or sim card? |  |  |  |  |  |
| … important phone numbers |  |  |  |  |  |

**Table S2:** Mother and Child Health Centre/recruitment site

| Characteristic |  | | The intervention group *n* = | | The control group *n* = | | *P-*value |
| --- | --- | --- | --- | --- | --- | --- | --- |
|  | *n* | (%) | *n* | (%) | *n* | (%) |  |
| Site 1 |  | |  |  |  |  |  |
| Site 2 |  |  |  |  |  |  |  |
| Site 3 |  |  |  |  |  |  |  |
| Site 4 |  |  |  |  |  |  |  |
| Site 5 |  |  |  |  |  |  |  |
| Site 6 |  |  |  |  |  |  |  |
| Site 7 |  |  |  |  |  |  |  |
| Site 8 |  |  |  |  |  |  |  |
| Site 9 |  |  |  |  |  |  |  |
| Site 10 |  |  |  |  |  |  |  |
| Site 11 |  |  |  |  |  |  |  |
| Site 12 |  |  |  |  |  |  |  |
| Site 13 |  |  |  |  |  |  |  |
| Site 14 |  |  |  |  |  |  |  |
| Site 15 |  |  |  |  |  |  |  |
| Site 16 |  |  |  |  |  |  |  |
| Site 17 |  |  |  |  |  |  |  |
| Site 18 |  |  |  |  |  |  |  |
| Site 19 |  |  |  |  |  |  |  |

# References

1. Henriksen L, Flaathen EM, Angelshaug J, Garnweidner-Holme L, Smastuen MC, Noll J, et al. The Safe Pregnancy study - promoting safety behaviours in antenatal care among Norwegian, Pakistani and Somali pregnant women: A study protocol for a randomized controlled trial. BMC Public Health. 2019;19(1):724.

2. Skevington SM, Lotfy M, O'Connell KA. The World Health Organization's WHOQOL-BREF quality of life assessment: Psychometric properties and results of the international field trial. A Report from the WHOQOL Group. Qual Life Res. 2004;13(2):299-310.

3. McFarlane J, Malecha A, Gist J, Watson K, Batten E, Hall I, et al. An Intervention to Increase Safety Behaviors of Abused Women: Results of a Randomized Clinical Trial. Nurs Res. 2002;51(6):347-54.

4. McFarlane J, Malecha A, Gist J, Watson K, Batten E, Hall I, et al. Increasing the safety-promoting behaviors of abused women. The American journal of nursing. 2004;104(3):40-50; quiz -1.

5. Ford-Gilboe M, Wathen CN, Varcoe C, MacMillan HL, Scott-Storey K, Mantler T, et al. Development of a brief measure of intimate partner violence experiences: The Composite Abuse Scale (Revised)-Short Form (CASR-SF). BMJ Open. 2016;6(12):e012824.

6. Rabin RF, Jennings JM, Campbell JC, Bair-Merritt MH. Intimate partner violence screening tools: A systematic review. American journal of preventive medicine. 2009;36(5):439-45 e4.

Signed:

Name, date and signature

Eva Marie Engebakken Flaathen 19.12 2020
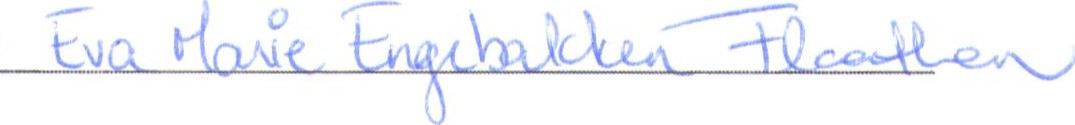


Lena Henriksen 19.12 2020


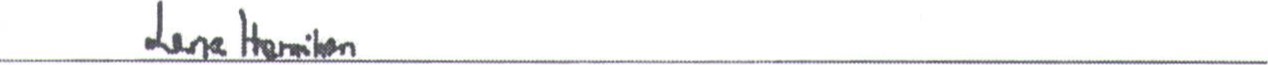


Milada Småstuen 19.19 2020

Mirjam Lukasse 19.12 2020


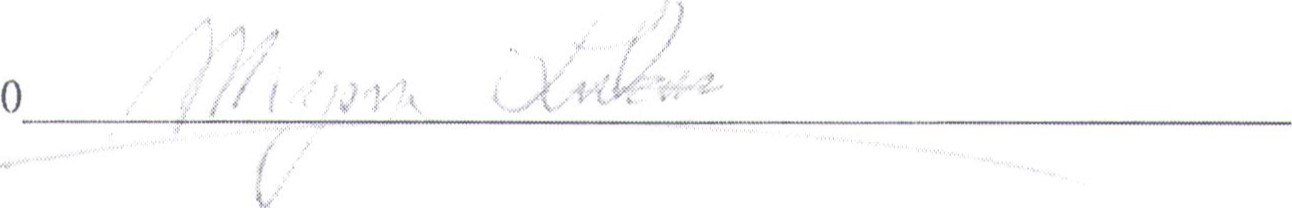

Supplement: Supplementary file 1 — Additional file 1. [file 12884_2022_4400_MOESM1_ESM.docx]
