# Supplementary material for: Safe Pregnancy intervention for intimate partner violence: a randomised controlled trial in Norway among culturally diverse pregnant women
Source: BMC Pregnancy Childbirth. 2022 Feb 21;22:144. doi: 10.1186/s12884-022-04400-z (PMC8862262; doi:10.1186/s12884-022-04400-z)
Supplement: Supplementary file 2 — Additional file 2. [file 12884_2022_4400_MOESM2_ESM.docx]

**Supplementary Table 1** Characteristics of responders and non-responders at follow-up (*N* = 317)

|  | **Total**  *N* = 317 (%) | **Responders**  **Q2**  *N* = 251 (%) | **Non-responders Q2**  *N* = 66 (%) | ***P-*value** |
| --- | --- | --- | --- | --- |
|  |  | **Mean (SD)** | **Mean (SD)** |  |
| **Age** | 317 (100) | 31.5 (4.75) | 31.4 (5.40) | 0.837 |
| **Gestational age at baseline** | 317 (100) | 28.8 (6.83) | 28.1 (5.67) | 0.425 |
|  | **n (%)** | **n (%)** | **n (%)** |  |
| **Civil status** |  |  |  | 0.266 |
| Married/cohabiting | 282 (88.9) | 224 (89.2) | 58 (87.9) |  |
| Single | 23 (7.3) | 16 (6.4) | 7 (10.6) |  |
| Missing | 12 (3.8) | 11 (4.4) | 1 (1.5) |  |
| **Education** |  |  |  | 0.294 |
| ≤ High school | 115 (36.3) | 89 (35.4) | 26 (39.4) |  |
| College/university  < 4 years | 91 (28.7) | 77 (30.7) | 14 (21.2) |  |
| College/university  ≥ 4 years | 109 (34.4) | 83 (33.1) | 26 (39.4) |  |
| Missing | 2 (0.6) | 2 (0.8) | 0 (0) |  |
| **Employment status** |  |  |  | 0.286 |
| Working/studying | 240 (75.8) | 193 (76.9) | 47 (71.2) |  |
| Unemployed | 75 (23.6) | 56 (22.3) | 19 (28.8) |  |
| Missing | 2 (0.6) | 2 (0.8) | 0 (0) |  |
| **Joint family income last year** |  |  |  | 0.378 |
| ≤ NOK 599,000 | 72 (22.7) | 57 (22.7) | 15 (22.7) |  |
| NOK 600–999,000 | 135 (42.7) | 109 (43.4) | 26 (39.4) |  |
| ≥ NOK 1,000,000 | 74 (23.3) | 60 (23.9) | 14 (21.2) |  |
| Do not know | 34 (10.7) | 23 (9.2) | 11 (16.7) |  |
| Missing | 2 (0.6) | 2 (0.8) | 0 (0) |  |
| **Mother tongue** |  |  |  | 0.178 |
| Norwegian | 235 (74.1) | 190 (75.7) | 45 (68.2) |  |
| Other | 80 (25.3) | 59 (23.5) | 21 (31.8) |  |
| Missing | 2 (0.6) | 2 (0.8) | 0 (0) |  |
| **Parity** |  |  |  | 0.366 |
| Nulliparous | 161 (50.8) | 124 (49.4) | 37 (56.1) |  |
| Multiparous | 154 (48.6) | 125 (49.8) | 29 (43.9) |  |
| Missing | 2 (0.6) | 2 (0.8) | 0 (0) |  |
| **Tobacco use** |  |  |  | 0.538 |
| Yes | 14 (4.4) | 12 (4.8) | 2 (3.0) |  |
| No | 303 (95.6) | 239 (95.2) | 64 (97.0) |  |
| **Negative experiences with alcohol consumption (woman)** |  |  |  | 0.211 |
| Yes | 47 (14.8) | 34 (13.5) | 13 (19.7) |  |
| No | 270 (85.2) | 217 (86.5) | 53 (80.3) |  |
| **Negative experiences with alcohol consumption (partner)** |  |  |  | 0.430 |
| Yes | 43 (13.6) | 36 (14.3) | 7 (10.6) |  |
| No | 274 (86.4) | 215 (85.7) | 59 (89.4) |  |
| **Maternal and child health centre** |  |  |  | 0.312 |
| Small (< 100) | 39 (12.3) | 30 (12.0) | 9 (13.6) |  |
| Medium (100–300) | 70 (22.1) | 60 (23.9) | 10 (15.2) |  |
| Large (> 300) | 208 (65.6) | 161 (64.1) | 47 (71.2) |  |
| **Video** |  |  |  | 0.233 |
| Intervention | 157 (49.5) | 120 (47.8) | 37 (56.1) |  |
| Control | 160 (50.5) | 131 (52.2) | 29 (43.9) |  |

**Supplementary Table 2** Abuse Assessment Screen at baseline: Q2 responders and non-responders (*N* = 317)

| AAS 1–5 | Total  *N* = 317 | Responders  *n* = 251 | Non-responders  *n* = 66 | *P-*value |
| --- | --- | --- | --- | --- |
| Fear |  |  |  | 0.817 |
| Never | 128 (40.4) | 99 (39.4) | 29 (43.9) |  |
| Previously | 149 (47.0) | 119 (47.4) | 30 (45.5) |  |
| Recent | 27 (8.5) | 23 (9.2) | 4 (6.1) |  |
| Previous and recent | 13 (4.1) | 10 (4.0) | 3 (4.5) |  |
| Afraid of partner |  |  |  | 0.147 |
| Never | 126 (39.7) | 93 (37.1) | 33 (50.0) |  |
| Previous | 176 (55.5) | 147 (58.6) | 29 (43.9) |  |
| Recent | 12 (3.8) | 9 (3.6) | 3 (4.5) |  |
| Previous and recent | 2 (0.6) | 1 (0.4) | 1 (1.5) |  |
| Missing | 1 (0.3) | 1 (0.4) | 0 (0) |  |
| Emotional IPV |  |  |  | 0.089 |
| No | 98 (30.9) | 78 (31.1) | 20 (30.3) |  |
| Previous | 200 (63.1) | 162 (64.5) | 38 (57.6) |  |
| Recent | 11 (3.5) | 6 (2.4) | 5 (7.6) |  |
| Previous and recent | 7 (2.2) | 4 (1.6) | 3 (4.5) |  |
| Missing | 1 (0.3) | 1 (0.4) | 0 (0) |  |
| Physical IPV |  |  |  | 0.229 |
| No | 222 (70.0) | 172 (68.5) | 50 (75.8) |  |
| Previous | 87 (27.4) | 74 (29.5) | 13 (19.7) |  |
| Recent | 4 (1.3) | 2 (0.8) | 2 (3.0) |  |
| Previous and recent | 4 (1.3) | 3 (1.2) | 1 (1.5) |  |
| Sexual IPV |  |  |  | 0.006 |
| No | 260 (82.0) | 197 (78.5) | 63 (95.5) |  |
| Previous | 56 (17.7) | 53 (21.1) | 3 (4.5) |  |
| Recent | 1 (0.3) | 1 (0.4) | 0 (0) |  |
| Previous and recent | 0 (0) | 0 (0) | 0 (0) |  |

**Supplementary Table 3** Maternal and child health centres (*N* = 18)*

| Characteristic |  | Intervention group *n* = 120 | Control group *n* = 131 | *P-*value |
| --- | --- | --- | --- | --- |
|  | *N* (%) | *n* (%) | *n* (%) | 0.61 |
| Site 1 | 22 (8.8) | 13 (10.8) | 9 (6.9) |  |
| Site 2 | 14 (5.6) | 10 (8.3) | 4 (3) |  |
| Site 3 | 20 (8.0) | 10 (8.3) | 10 (7.6) |  |
| Site 4 | 16 (6.4) | 6 (5) | 10 (7.6) |  |
| Site 5 | 8 (3.2) | 5 (4.2) | 3 (2.3) |  |
| Site 6 | 19 (7.5) | 7 (5.8) | 12 (9.2) |  |
| Site 7 | 16 (6.4) | 8 (6.7) | 8 (6.1) |  |
| Site 8 | 19 (7.5) | 9 (7.5) | 10 (7.6) |  |
| Site 9 | 9 (3.6) | 2 (1.7) | 7 (5.3) |  |
| Site 10 | 1 (0.4) | 0 (0) | 1 (0.8) |  |
| Site 11 | 6 (2.4) | 1 (0.8) | 5 (3.8) |  |
| Site 12 | 22 (8.8) | 12 (10) | 10 (7.6) |  |
| Site 13 | 26 (10.3) | 14 (11.7) | 12 (9.2) |  |
| Site14 | 1 (0.4) | 0 (0) | 1 (0.8) |  |
| Site 15 | 16 (6.4) | 7 (5.8) | 9 (6.9) |  |
| Site 16 | 5 (2.0) | 3 (2.5) | 2 (1.5) |  |
| Site 17 | 5 (2.0) | 2 (1.7) | 3 (2.3) |  |
| Site 18 | 26 (10.3) | 11 (9.2) | 15 (11.5) |  |

*One of the 19 MCHCs did not report any follow-up cases due to a short recruitment period.
